# Knowledge-based annotation of small molecule binding sites in proteins

**DOI:** 10.1186/1471-2105-11-365

**Published:** 2010-07-01

**Authors:** Ratna R Thangudu, Manoj Tyagi, Benjamin A Shoemaker, Stephen H Bryant, Anna R Panchenko, Thomas Madej

**Affiliations:** 1 National Center for Biotechnology Information, 8600 Rockville Pike, Building 38A, Bethesda, MD 20894 USA

## Abstract

**Background:**

The study of protein-small molecule interactions is vital for understanding protein function and for practical applications in drug discovery. To benefit from the rapidly increasing structural data, it is essential to improve the tools that enable large scale binding site prediction with greater emphasis on their biological validity.

**Results:**

We have developed a new method for the annotation of protein-small molecule binding sites, using inference by homology, which allows us to extend annotation onto protein sequences without experimental data available. To ensure biological relevance of binding sites, our method clusters similar binding sites found in homologous protein structures based on their sequence and structure conservation. Binding sites which appear evolutionarily conserved among non-redundant sets of homologous proteins are given higher priority. After binding sites are clustered, position specific score matrices (PSSMs) are constructed from the corresponding binding site alignments. Together with other measures, the PSSMs are subsequently used to rank binding sites to assess how well they match the query and to better gauge their biological relevance. The method also facilitates a succinct and informative representation of observed and inferred binding sites from homologs with known three-dimensional structures, thereby providing the means to analyze conservation and diversity of binding modes. Furthermore, the chemical properties of small molecules bound to the inferred binding sites can be used as a starting point in small molecule virtual screening. The method was validated by comparison to other binding site prediction methods and to a collection of manually curated binding site annotations. We show that our method achieves a sensitivity of 72% at predicting biologically relevant binding sites and can accurately discriminate those sites that bind biological small molecules from non-biological ones.

**Conclusions:**

A new algorithm has been developed to predict binding sites with high accuracy in terms of their biological validity. It also provides a common platform for function prediction, knowledge-based docking and for small molecule virtual screening. The method can be applied even for a query sequence without structure. The method is available at http://www.ncbi.nlm.nih.gov/Structure/ibis/ibis.cgi.

## Background

The physical interactions between proteins and other molecules in protein crystal structures provide crucial insights into protein function. It is precisely these structures that enable researchers to study interactions in atomic detail, and find out, for example, how a specific mutation in a protein affects its function, or how a few atom modifications in a small molecule might lead to a more effective drug. With the large number of available crystal structures (nearly 60,000 currently in the RCSB Protein Data Bank), it is of great importance to improve the tools available for study of these interactions.

Moreover, a powerful method of inference can be used to predict function and interactions. It is based on the observation that homologous proteins have similar functions and often interact with their small molecules in a similar manner. Thus it is possible to infer protein-small molecule interactions even if there are no crystal structures available for a particular protein of interest, as long as there are structures of sufficiently close homologs. Recent estimates suggest that the majority of Entrez Protein sequences have homologs with a known structure [1,2], thereby providing a reasonable chance to find relevant interactions via structures for protein sequences.

Homology inference methods, although powerful, have certain limitations. Common descent does not necessarily imply similarity in function or interactions; and annotations transferred from one protein to a homolog may result in incorrect functional or interolog assignment at larger evolutionary distances [3-6]. To verify and guide annotations, it is often essential to ensure close evolutionary relationships, and at the same time characterize the details of interactions in terms of binding site similarity. Current binding site prediction methods can be subdivided into several major categories: those which use evolutionary conservation of binding site motifs [7-9], those which use information about a structure of a complex [10-12], and docking and other methods [13,14]. Structure-based methods use detailed knowledge of the protein structure to identify binding sites on the basis of the physico-chemical properties of individual residues, their electrostatic contribution, and their location in the 3D structure [15-26].

A number of methods and servers have been developed for predicting protein function by identifying similarities in sequence and structural features of binding pockets in homologous proteins, or evolutionary constraints on residues [27], or by using threading and other approaches [20,28-39]. The main goal of these methods is to provide functional annotation for proteins out to the most distant homology relationships. *FINDSITE *[40], for example, looks for structural templates with bound small molecules for a query protein using threading. The templates are superimposed and the centers of mass of the bound small molecules are clustered to annotate putative binding sites on the query. Threading based methods, although capable of recognizing distant functional relations, are limited by the complexity of model building and low reliability of function transfer associated with distant homology [41,42].

*Firestar *[31] predicts functionally important residues based on PSI-BLAST [43] alignments between the query sequence and structures with functional information derived from the PDB and the Catalytic Site Atlas [44]. *PHUNCTIONER *[20] uses sequence profiles based on clustered sequences with matching GO [45] terms; potential binding sites are detected from sequence conservation. This method is capable of inferring the location of highly conserved small molecule binding sites, but might be questionable if the conservation of sites is caused by factors other than binding.

Transitive annotation of small molecule binding sites is also possible by detection of functional domains in the query protein sequence through BLAST heuristics and mapping the functionally important residues and/or features from the domain family members [30,46].

There are a few other methods that directly detect small molecule binding sites via geometric analysis of protein structures. These methods include LIGSITE^csc ^[29], CAST [47], PASS [48], SURFNET [49], SCREEN [50], and ConCavity [51]. All of these algorithms attempt to identify solvent-accessible pockets formed by surface residues on the protein, and to rank those pockets (for example by volume), in order to assign the most highly ranked pockets as the predicted/putative small molecule binding sites. LIGSITE^csc^, SURFNET, and ConCavity use a more complex ranking function that takes into account residue conservation of binding site residues. These geometric methods are reasonably accurate, achieving success rates of 60-70% in correctly identifying small molecule binding sites. In their evaluation of LIGSITE^csc^, the authors showed that their algorithm outperformed the other three methods on a test set of 48 structures [29]. The SCREEN method identifies binding sites geometrically, and also computes feature vectors that are used by machine learning techniques. SCREEN is included in a suite of powerful modeling tools for functional annotation [52]

Recently we have developed a new database and method called "IBIS" (Inferred Biomolecular Interaction Server [53], http://www.ncbi.nlm.nih.gov/Structure/ibis/ibis.html) which enables researchers to conveniently study biomolecular interactions that have been observed in protein structures and through inference by homology to formulate predictions/hypotheses for biomolecular interactions, even if the data for specific biomolecules is not available. Therefore, IBIS can be considered a resource for functional annotation of proteins that have relevant homologs in the PDB [54]. An input protein sequence may or may not have a structure itself; if not, it is assigned to the most closely related structure(s) using BLAST. IBIS can identify and infer a protein's interaction partners together with the locations of the corresponding binding sites on the protein query. It provides annotations of binding sites for proteins, small molecules (chemicals), nucleic acids, peptides and ions. In this paper we describe the method used in IBIS to annotate protein-small molecule interactions. To ensure biological relevance of binding sites, IBIS clusters similar binding sites found in homologous proteins based on conservation of sequence and structure of the binding site residues. Binding sites which appear evolutionarily conserved among non-redundant sets of homologous proteins are given higher priority. Additionally, binding site clusters are validated by comparing them with available binding site annotations from a manually curated subset of the CDD database [55,56], and sites with non-biological small molecules are excluded. After binding sites are clustered, position specific score matrices (PSSMs) are constructed from the corresponding binding site alignments. Together with other measures, the PSSMs are subsequently used to rank binding sites to assess how well they match the query, and to gauge the biological relevance of binding sites with respect to the query.

A critical difference between our method and others is that IBIS pays particular attention to ensuring the biological relevance of binding sites, and homology between the unknown query sequence and the known structures of protein complexes. Our method might miss some remote similarities which could be detectable, for example by FINDSITE, but in exchange IBIS's top ranked annotations should be considered highly reliable. Unlike other methods, IBIS does not filter out similar structures to speed up the search process, but accounts for all structures so that interesting small molecule binding complexes are easily accessible. Our method derives the actual binding sites from observed structures, and groups them to account for variations in the binding site residues due to differences in small molecule size and conformations. This is essential for proteins which are important drug targets, as they have often been co-crystallized with a great variety of inhibitors. The clustering (grouping) of binding sites by similarity is very important because it identifies the distinct binding modes and allows for an easier interpretation of the results, despite the great growth in the amount of structure data over the last several years. As we have shown, it is possible to do the clustering automatically and in a biologically meaningful way.

## Results

### Annotation of protein chains with observed and inferred binding sites

**Figure 1:**
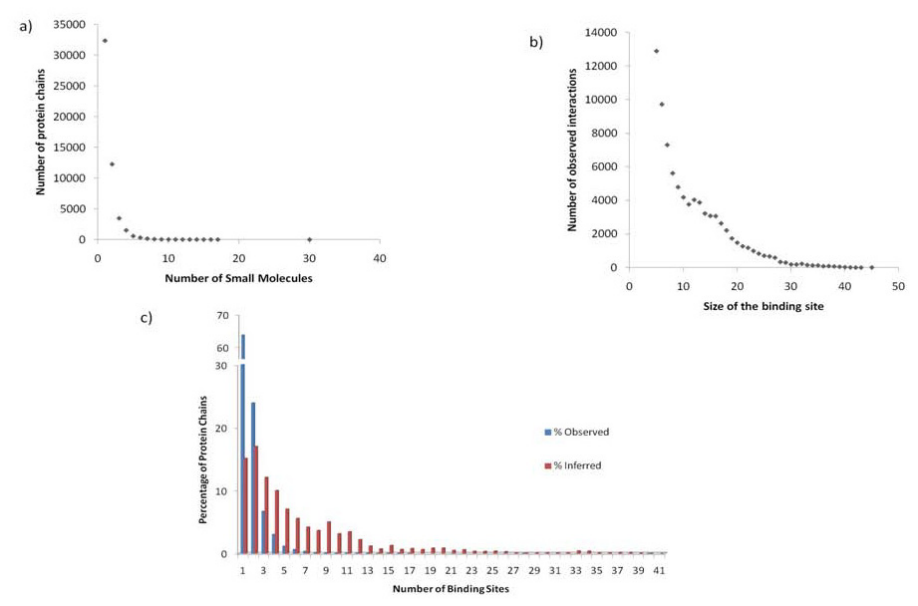
**Statistics of small molecules and their binding sites observed in protein structure complexes**. a) Number of small molecules and binding sites observed per protein chain, b) size of the observed binding sites, c) histogram showing the number of observed and inferred binding sites with plotted versus the fraction (%) of protein chains having these sites.

There are about 28000 PDB entries with observed small molecule binding sites and about 56000 protein chains. The total number of observed small molecule binding sites is about 91000 and approximately 67000 of these represent biologically relevant small molecules(i.e. around 24000 small molecules represent crystallization agents). Small molecule binding is a specific feature that plays a crucial role in the protein function. About 64% of protein chains in the PDB bind to a single small molecule and 95% bind to no more than four small molecules (Figure 1a). Likewise, the binding site pockets are rather small compared to the size of their functional domains. The binding sites are usually less than 25 residues and 55% of the binding sites in the current study are smaller than 10 residues (Figure 1b). Our algorithm inferred binding sites for 92000 protein chains and the overall average number of binding site clusters inferred per chain is 6.5 (Figure 1c) whereas the average number of biologically relevant binding site clusters inferred per chain is about 4.

One of the important features of this method is that it does not exclude redundant sequences bound to different small molecules. For example, to account for all specific interactions of various drugs targeting the Kinase ATP binding site, it is imperative to consider all the protein sequences even if they are identical.

We validated the IBIS method by comparing the obtained annotations to the manually curated CDD annotations and to other different methods which use geometry of binding pockets and/or sequence conservation of binding sites. It should be mentioned that since the IBIS method is based on different types of structural evidence, the notion of false positives might not be valid in many cases.

### Validation of the IBIS method using the Conserved Domain Database

To test the ability of our method to successfully infer the biologically relevant binding sites, a validation procedure was implemented using the manually curated Conserved Domain Database (CDD) [56] alignments and the functional features recorded in it as a standard of truth. Manually curated functional site annotations in CDD have been extracted from the published literature or derived from manual interpretation of individual three-dimensional structures. Altogether 49% of the proteins with observed small molecule binding sites have CDD small molecule binding site conserved annotations whereas over 55% of the proteins with inferred binding sites have at least one site overlapping with CDD annotated binding site annotation.

**Figure 2:**
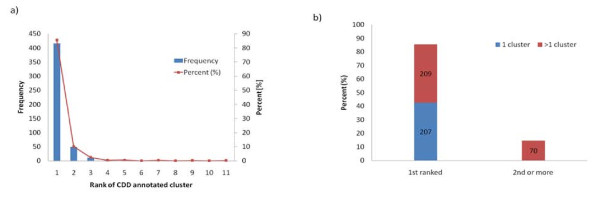
**Biological validity of the IBIS inferred binding sites**. a) Histogram showing the frequency of protein chains as function of their biological relevancy as suggested by overlap of the inferred binding sites with CDD conserved feature annotation. b) Percentage of proteins with their inferred sites having their 1^st ^and 2^nd ^rank clusters with CD annotations; 165 proteins have only one predicted site.

In our analysis we used the CDD release 2.16 containing 4092 protein chains. We chose representative protein chains purged at the 25% sequence identity level. In this jackknifing experiment, the query protein and its identical homologs are omitted from clustering. Altogether 486 representative chains had at least one structurally similar non-identical homolog which had observed protein-small molecule binding sites. Figure 2a shows how well our method can retrieve the CDD annotated binding sites at the top ranks by calculating the fraction of true positives (sensitivity) or percentage of correctly annotated binding sites (overlap between CDD and IBIS annotated binding sites should be at least 50%). For 207 of these there was only one inferred binding site (cluster) detected, and by default these will always be ranked first. There remain 279 examples which have at least two IBIS binding sites, 209 (75%) of these were ranked first, and 49 were ranked second, so that 258 (92%) were ranked either first or second (Figure 2b).

Since there are a number of proteins which do not have CDD annotations, IBIS inferred binding sites may be biologically relevant in these cases.

### Validation of ranking scheme: discriminating between biological and non-biological chemicals

We used the same set of protein queries (604 chains) to evaluate our method using structures which contained both biological and non-biological small molecules (see Additional file 1 Table S1). Our goal is to assess how well our ranking scheme distinguishes between the two groups of binding sites: those containing biological versus non-biological small molecules. If all the bound small molecules in an inferred binding site are non-biological, it is deemed as non-biological. To address this, we applied a linear discriminant analysis which constructs a discriminant function that divides the parameter space into regions so as to separate the groups as distinctly as possible. The method computes the posterior probability of group membership for each observation, and assigns the observation to the group that has the highest probability. As a result, a classification matrix is produced, which gives the fraction of observations correctly assigned to each group by the discriminant function. In our case, a good classification would be quantified by high fractions for both correctly predicted biological binding site clusters and correctly predicted non-biological binding site clusters. We found that our method correctly classifies 87% of biological clusters and 85% of non-biological clusters.

### Validation of IBIS method by comparison with - geometric methods

To further validate the prediction ability of our method we compared it with several widely used geometry and energy-based approaches discussed in a recent study [57] which includes LIGSITEcsc [29], PASS [48], Q-SiteFinder [28], Surfnet [49]. We used 44 out of 48 proteins from this paper which have structure homologs with at least 30% sequence identity and also have both small molecule-bound and unbound structures.

For each method tested, the top ranked predicted sites for the unbound structure are compared with the observed binding sites in the bound structure of a protein-small molecule complex of a homolog. Table 1 shows the sensitivity of retrieval of the true observed sites at the top three ranks. To measure the sensitivity of retrieval of bound structures at different levels of similarity between the unbound query and bound structure from the database, we selected from the test set only those unbound-bound pairs which are within a given similarity range (no more than 80, 90, or 100% identity) and denoted them IBIS_80_, IBIS_90 _and IBIS_100_. For example, the IBIS_90 _dataset contains unbound query proteins for which the average sequence identity between the unbound protein and members of the binding site clusters containing the bound homolog is no more than 90%. It is difficult if not impossible to define false positives in our case since there are many binding site clusters which are biologically relevant (for example have a significant overlap with the manually curated CDD functional annotations) but at the same time do not match the binding site of the bound form of the protein from the test set. This happens if, for example, there are multiple binding sites/pockets in the protein which bind different small molecules and have distinct functions. As can be seen from this table IBIS performance is similar to the LIGSITE^cs ^method which uses sequence conservation and reaches about 72% sensitivity. Overall we found that a total of 31 proteins (70%) from the test set have at least one of their IBIS predicted sites overlap with CDD binding site annotation. This suggests that IBIS successfully uses the knowledge of the structure complexes of homologs to predict and rank the relevant sites. The complete details of the prediction results can be seen in Additional file 1 Table S2.

**Table 1:** Prediction sensitivity (%) of the top three predictions by different geometric approaches and their comparison to IBIS.

Method*	Top1	Top2	Top3
IBIS_100_	73	89	89
IBIS_90_	75	91	91
IBIS_80_	72	88	88
LIGSITEcs	71	79	85
PASS	58	67	75
Q-SiteFinder	52	60	75
SURFNET	42	58	62

*IBIS_100_, IBIS_90_, IBIS_80 _dataset contains unbound query proteins for which the average sequence identity between the unbound protein and members of the binding site clusters containing the bound homolog is no more than 100%, 90% and 80% respectively.

All of these approaches, although they perform reasonably well, are limited by the requirement of differentiating true positives from false positives. Introducing sequence conservation need not necessarily improve the prediction accuracy and could be a source of error, leading to over prediction of the binding site area [58]. IBIS on the other hand predicts only a handful of small molecule binding sites with high probability of being biologically relevant. On average our method predicts 4 'biologically relevant' binding sites per protein chain and over half of all predicted sites map to CDD curator annotations.

### Knowledge-based docking using IBIS, an example

To demonstrate the effectiveness of IBIS as a knowledge-based prediction system, we compared our method with an established reverse docking approach. Cai and coworkers [59] employed a reverse docking method to find a potential target protein for a natural product: *N*-*trans*-caffeoyltyramin in the genome of *Helicobacter pyroli*. Initially all potential binding proteins of *N*-*trans*-caffeoyltyramin were screened from a database of potential drug targets with known structures from the Protein Data Bank using the reverse docking approach TarFisDock [60]. Only two proteins from the *H.pyroli *genome were found by the TarFisDock method: diaminopimelate decarboxylase (DC) and Peptide deformylase (PDF). After enzymatic validation, only the PDF protein was found to be a probable drug target. The crystal structure complex of *N*-*trans*-caffeoyltyramin with PDF suggested a highly selective binding in the PDF binding pocket.

**Figure 3:**
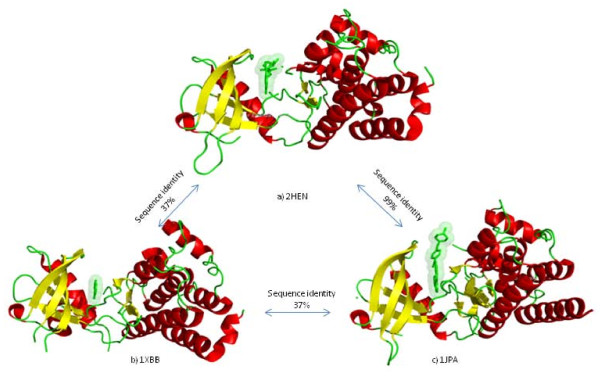
**Tyrosine kinase homologoues with varying degrees of sequence conservation with different small molecules in their ATP-binding pocket**. (a) Ephb2 Receptor Kinase domain with ADP. (b) Syk Tyrosine Kinase Domain in complex with Gleevec. (c) Ephb2 Receptor Tyrosine Kinase with Adenine.

We attempted to identify the binding sites of *N*-*trans*-caffeoyltyramin on the PDF protein sequence. The closest homolog for *H.pyroli *PDF is *P.aeruginosa *PDF which has 45% sequence identity and has been used as a template for inferring the interactions by IBIS. The top ranked and highly conserved inferred binding site of P.aeruginosa PDF when mapped onto H.pyroli PDF is in complete agreement with the native/experimentally determined binding site of the *N*-*trans*-caffeoyltyramin - PDF complex (Figure 3).

## Discussion

**Figure 4:**
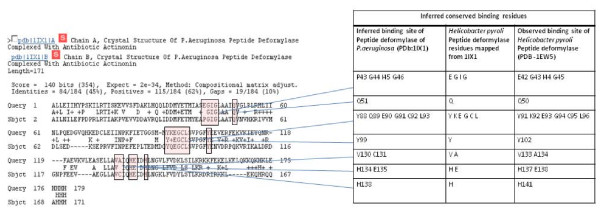
**Mapping of the inferred binding site**. Inferred binding site of peptide deformylase *P.aeruginosa *(PDB:1IX1) mapped onto the sequence of *Helicobacter pyroli *and its agreement with the observed binding site in *N*-*trans*-caffeoyltyramin-PDF complex (PDB: 1EW5). MMDB residue numbering is used which starts from the beginning of the corresponding GenBank protein sequence.

A researcher interested in the function of a specific protein will usually be concerned not only with the availability of any functional annotation, but also with the reliability of such information. The most reliable source is experimental data on the protein function but despite the growth of the protein sequence and structure databases, there remains only a small fraction of proteins whose functions have been experimentally characterized. In this paper we present a method which provides the information on protein function annotation through the identification of protein binding sites. The current approach attempts to interlink sequence conservation with structural diversity in deciphering protein function. We specifically focus on protein small molecule binding sites and their biological relevance for protein function. Our method derives the actual binding sites from the structures of all the homologs and groups them based on sequence and structural similarity. For example, to account for all specific interactions of various drugs targeting the Kinase ATP binding site (see Additional file 1 Table S3), it is imperative to consider all the protein sequences even if some of them are identical. Such grouping ensures their biological relevance and at the same time accounts for variations in the binding site residues due to differences in small molecule sizes and conformations. By using all available structures of close homologs, IBIS provides a great opportunity for analyzing the diversity of binding modes. Figure 4, for example, shows the conserved tyrosine kinase fold with varying degrees of sequence similarity but sharing a highly conserved ATP binding site occupied by different small molecules.

Recently, it was estimated that over two-thirds of all protein sequences in the GenBank database have at least one structure homolog [1,2]. As the on-going structural genomics initiative continues to close the sequence-structure gap, our method might be very useful for annotating proteins with unknown function and structure. Moreover, the location of putative binding sites provides guidance for the protein docking methods for drug design. We have assessed the reliability of our method by direct comparison with the binding site annotations from literature and manual curation and have shown that in the great majority of cases, the method detects and ranks the manually annotated binding site cluster at the first or second rank. This is achievable for a number of reasons, such as using a sufficient level of similarity between the unknown query and its homologs with the known binding sites, accurate clustering of small molecule binding sites using a reasonable similarity measure, and applying a deliberately designed ranking scheme that distinguishes the non-biological from the biologically relevant binding sites.

We have also compared our method with several widely used geometry and energy-based approaches to predict small molecule binding sites. As we have shown, the performance of our prediction method is very similar to popular geometric approaches. Moreover, one of the advantages is that our method can be applied even for a query sequence without structure, which is not the case for those binding site prediction methods which explicitly rely on the specific features of binding pocket geometry.

Using remote homology for functional inference is often based on the general assumption that there is a negative correlation between small molecule binding site similarity and overall sequence similarity. However, small molecule binding site similarity is much more complicated with many examples of strikingly similar binding sites with low (<30%) overall sequence identity and also very weakly similar binding sites with high overall sequence identity [61]. Likewise, the similarities of small molecule binding sites across different protein folds, although providing new insights, leads to new challenges in deciphering the functional relevance. Large-scale automated function prediction methods are often limited by the lack of sufficient understanding of biological function and also by the quality of structure data. Hence, through the IBIS approach, we strive to limit the false positive rate by employing a conservative sequence similarity threshold of at least 30% over the structurally superimposed regions of homologs. It is often possible that the protein-small molecule crystal state may correspond to a global minimum of free energy where biologically relevant interactions are difficult to distinguish from non-specific contacts. For example, a recent estimate suggests some 20% of dimeric structures in PDB may be crystallization artifacts [62]. The elaborate scoring scheme of our method based on recurrence and evolutionary conservation, along with the list of non-biological small molecules, tends to de-emphasize the artifactual interactions and ranks such sites near or at the bottom of the list.

Furthermore, the chemical properties of small molecules bound to the inferred binding sites can be used as a starting step in small molecule virtual screening. The PubChem compound database [63] mapping of IBIS small molecules accomplishes a preliminary step in small molecule virtual screening by clustering the similar chemicals into structurally unique compounds. The functional groups of the small molecules binding in a common binding site of evolutionarily related proteins are likely conserved. Recently it was shown that sequence and structure conservation of the binding site residues contacting these anchor functional groups is significantly higher than those contacting variable regions [40]. IBIS, thus provides a common platform for function prediction, knowledge-based docking and also for small molecule virtual screening.

## Conclusions

Finding small molecule binding sites that specify protein function is of great importance in drug development. Here we proposed a method to decipher the function of an unknown protein by interlinking sequence conservation with structural diversity of its homologs. To facilitate validation of the inferred binding sites from homologs, we developed an elaborate scoring scheme that can accurately distinguish biologically relevant sites. The method has been implemented as a web server, IBIS (Inferred Biomolecular Interaction Server - http://www.ncbi.nlm.nih.gov/Structure/ibis/ibis.cgi) to facilitate accurate, efficient and high-throughput function prediction.

## Methods

We used the NCBI Molecular Modeling Database (MMDB) [64] as a source of data on protein complexes. The automated MMDB processing of PDB files includes steps such as deposition of the protein sequences into GenBank [65], deposition of small molecules into PubChem [63], addition of corresponding links to these databases in the MMDB records, also links to citations and references in PubMed, and Entrez indexing for quick searching.

Below we describe different steps of processing, including defining observed interactions from structures, inferred interactions from homologs, clustering binding sites and their ranking in terms of biological relevance with respect to the query protein.

### Defining observed interactions

In the current release of the Molecular Modeling Database (MMDB) [64], there are about 28000 entries with bound small molecules. The resulting 39000 small molecules are bound to about 56000 protein chains in total. A small molecule is defined as any non-polypeptide, non-nucleic acid molecule in the structure complex or any molecule with a sufficient number of non-standard amino acid/nucleic acids and without an assigned GenBank identifier from NCBI. All the small molecules are standardized in the PubChem database [63] and have valid substance and compound identifiers. In this work we do not consider small molecules that are smaller than 5 heavy atoms or those having molecular weight outside the range of 70-800 Da. Small molecules such as metal ions often play role as crystallization agents, and therefore ions are not considered in this paper.

The filters by atom count and molecular weight only partially remove non-biological small molecules (i.e. buffers, salts, detergents, solvents, and ions added for the purpose of crystallization and/or purification). These non-biological molecules sometimes mimic natural small molecules and tend to bind in functional/active sites of proteins. For validation purposes we used a list of potential non-biological small molecules which has been collected from the literature (see Additional file 1 Table S1) [30,66,67].

We define a protein residue to be in contact with a small molecule if there is at least one (heavy) atom of the residue within 4.0Å of some atom from the small molecule. For most pairs of atoms, this threshold corresponds to the sum of their van der Waals radii plus a tolerance of about 0.5Å to allow for coordinate errors in structure determination. For manual curation of the Conserved Domain Database (CDD) a similar contact definition is used for defining protein-small molecule contacts. We retain only those protein-small molecule complexes which have at least five interacting protein residues. We define *"binding site" *as a set of residues on a given protein chain which are in contact with a given small molecule. Each MMDB entry is analyzed, and all pairs of biomolecules consisting of a protein chain and small molecule in contact with that chain are retained for further analysis.

It should be mentioned that a small molecule can be bound to a single domain or multiple domains which could come from more than one protein chain in the PDB record. Almost half of all the small molecules in the PDB are bound by more than one domain with <75% of all contacts to any single domain [66]. However, using domains as structural units would necessitate automatic domain decomposition methods in many cases [68,69], and the domain boundaries chosen could affect the results. To circumvent the potential technical difficulties in using domains as the structural unit in recording the observed/physical interactions, we use only complete protein chains for defining protein-small molecule interactions. Small molecules binding to multiple protein chains entail even more technical difficulties. For example, simultaneous superposition of multiple chains would need to be checked to ensure similarity of binding sites. Therefore, when multiple chains are involved in a binding site, if one of the chains includes 75% or more of the contacts, then we define only one binding site and assign it only to that particular chain. Otherwise, we define separate binding sites on each of the chains. The latter situation is relatively rare as only about 15% of the proteins in the current PDB release have small molecule interactions that fall into this category.

### Inferring interactions from homologs

**Figure 5:**
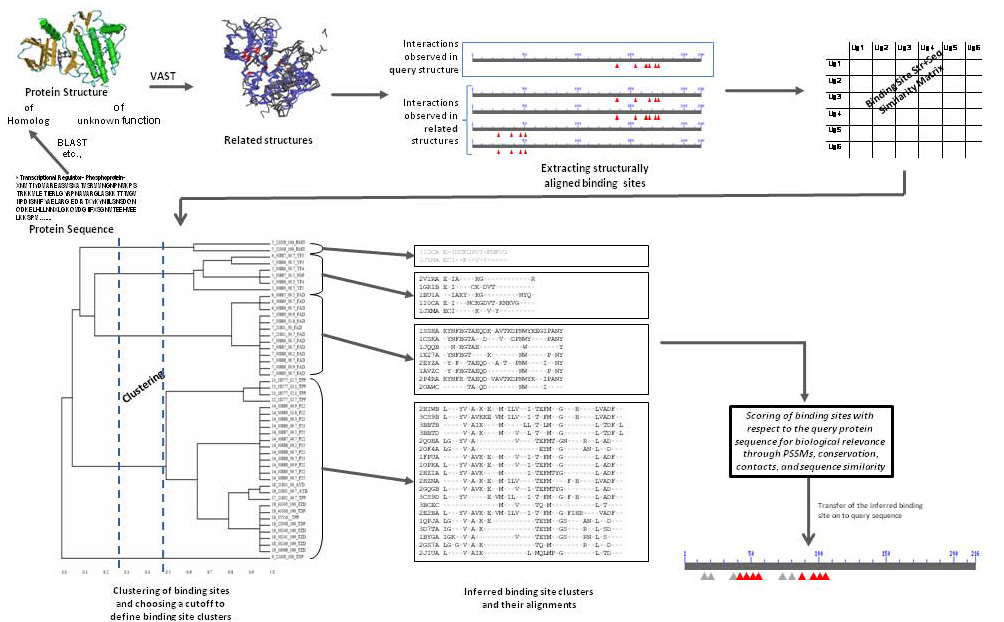
**Overview of the IBIS binding site annotation procedure**.

To ensure the biological relevance of binding sites, they are clustered and their sequence and structural similarity is assessed. An overview of the process is shown in Figure 5. Here are the important details concerning the main steps in the processing.

#### 1) Collecting homologs with bound small molecules

To infer interactions based on homology we collect proteins which are structurally similar to a given query protein and have at least 30% sequence identity to the query (we refer to them as "homologous structure neighbors"). Structure neighbors for all PDB/MMDB entries have been pre-computed by the VAST algorithm [70] and stored in the PubVast database. Then we retrieve observed interactions for all structure neighbors (including the query protein). No sequence redundancy filter is applied to remove structures because there are often many structures of the same protein with different bound small molecules, and we may wish to study any of these cases. Since the alignments may contain gaps, we retain only those instances where at least 75% of the residue contacts with the small molecule occur within the structure alignment footprint of the query and neighbor.

#### 2) Measuring binding site similarity

In order to cluster the binding sites of the homologous structure neighbors, it is necessary to construct their alignment and define a similarity measure. We construct the alignment between the structure neighbors *A *and *B *by composing the alignment from structure neighbor *A *to the query, with the alignment from the query to structure neighbor *B*. It is necessary to construct this alignment by composing through the query, because oftentimes the neighbors *A *and *B *will be more closely related to each other than to the query, in which case the "direct" alignment between them will be more extensive than the one through the query, and so it could include binding sites or interface residues that are not relevant to the query. To capture the similarity of the binding sites, the similarity measure includes both the structural equivalency and sequence similarity. The similarity score between two positions *i *and *j *of two binding sites is defined as:(1)

where *H *is the element of the BLOSUM62 matrix corresponding to the aligned amino acids in positions *i *and *j*; *Δ*_*ij *_is equal to 1 if two positions are aligned and 0 otherwise. ***θ ***is an additional weight of "+1" added to each structurally equivalent position. *w *is a gap penalty of "-4", to mimic the most unfavorable substitution score from BLOSUM62 matrix, which showed the best performance in our preliminary studies. The overall similarity score between two binding sites is calculated by summing up *S_ij _*over all positions in the gapped alignment. To facilitate comparison of scores from different alignments, the raw score is converted to a bit score with the statistical parameters λ and *K *previously defined in the BLOSUM scoring system.(2)

The similarity score is then converted into a conservation score *CS *by dividing by the maximum of the bit scores when the binding sites are scored against themselves.(3)

#### 3) Clustering of binding sites

Based on the calculated conservation score *CS*, the binding sites of the homologs are clustered using a complete-linkage clustering algorithm, which considers the distance between two clusters to be equal to the maximum distance between their members. A distance cutoff value to define the clusters is chosen using a free energy function defined previously. This function *F *is formulated to maximize the mean similarity of members within a cluster and minimize the complexity of the description provided by cluster membership [71].(4)

where *T *is the temperature factor, *S(i, j) *is the similarity score between binding site *i *and binding site *j *in each cluster, *C *represents a cluster, |*C*| is the number of binding sites in the cluster *C*, and *N *is the total number of binding sites clustered. The temperature *T *is a parameter (constant) that is chosen so as to correctly balance the energy-like and entropy-like terms in the function [71].

### Biological relevance of binding sites and their ranking with respect to the query protein

All binding site clusters are ranked in terms of their predicted biological relevance and similarity to the query. First we assess the evolutionary conservation of binding site clusters. Those sites which reoccur in diverse enough protein complexes are ranked higher. Clusters that have only one non-redundant member (after members with more than 90% identity are removed) are considered "singletons" and are not assigned any score (ranked at the bottom of the list). A "conservation score" is computed in order to measure the diversity of cluster members and how well the binding site is conserved across the homologs. To do this, positional conservation in the binding site multiple sequence alignment is calculated using the Shannon entropy measure with the Henikoff-Henikoff sequence weights. Sequence weights are estimated using the complete sequences of neighbors aligned with the query protein.

To account for evolutionary closeness of a given binding site cluster to the query we use the sequence-PSSM score and the average sequence identity between the query and all cluster members calculated over the whole structure-structure alignment (not just binding sites). A position specific score matrix (PSSM) is constructed based on the binding site multiple alignment using the implicit pseudo-count method of Gribskov, McLachlan and Eisenberg [72]. The aligned binding site region of the query protein is then scored against the PSSM and a sequence-PSSM score is calculated. A higher sequence-PSSM score points to a higher probability of this site being a biologically relevant site for the query.

To rank the larger interfaces more highly we also calculate the average number of interfacial contacts which the binding site makes in the complex of the corresponding homolog. All components of the ranking score are then normalized and all clusters are ranked with respect to the Z-scores. Any cluster with all members binding non-biological small molecules is disregarded.

The Z-score is calculated for each of these four corresponding terms (i.e. conservation score, PSSM-score, contact count, and percent sequence identity to query) in the ranking scheme by subtracting the mean value and dividing by the standard deviation obtained from the score distribution of other binding site clusters for a given query protein. The coefficients in front of each term in the ranking score were calculated empirically. The combined score is designed to rank the most biologically relevant sites at the top.(5)

## Authors' contributions

ARP, BAS, SHB, and TM conceived the project. RRT, MT, and BAS implemented the database and analysis programs. RRT, ARP, and TM wrote the paper. All authors contributed to the underlying ideas of the method and the analysis. All authors read and approved the final manuscript.

## Supplementary Material

Additional file 1 **Table S1: The most common non-biological small molecules found in protein structure complexes**. Table S2: Summary of the IBIS predictions and CDD annotation validation for the 44 bound and unbound structures used as test set to compare with existing geometric approaches. Table S3: Variety of small molecules binding in the ATP binding pocket of tyrosine kinase homologs. Click here for file
