# Supplementary material for: Knowledge-based annotation of small molecule binding sites in proteins
Source: BMC Bioinformatics. 2010 Jul 1;11:365. doi: 10.1186/1471-2105-11-365 (PMC2909224; doi:10.1186/1471-2105-11-365)
Supplement: Additional file 1 — Table S1: The most common non-biological small molecules found in protein structure complexes. Table S2: Summary of the IBIS predictions and CDD annotation validation for the 44 bound and unbound structures used as test set to compare with existing geometric approaches. Table S3: Variety of small molecules binding in the ATP binding pocket of tyrosine kinase homologs. [file 1471-2105-11-365-S1.DOCX]

Table S1: The most common non-biological ligands found in protein structure complexes

| Ligand Name | Frequency |
| --- | --- |
| SULFURIC ACID | 2747 |
| glycerol | 2395 |
| Phosphoric acid | 1072 |
| 558079_ALDRICH | 280 |
| MES solution | 251 |
| 2-mercaptoethanol | 219 |
| TROMETHAMINE | 194 |
| beta-D-glucose | 177 |
| DIETHYLENE GLYCOL | 159 |
| HEPES | 148 |
| TETRAETHYLENE GLYCOL | 144 |
| Hydrargyrum | 138 |
| hexylene glycol | 134 |
| pyrophosphate | 124 |
| Triglycol | 113 |
| CADMIUM | 101 |
| dimethyl sulfoxide | 93 |
| B-Octylglucoside | 92 |
| Pentaethylene glycol | 73 |
| CAC | 70 |
| bromide | 62 |
| Hexagol | 54 |
| p-Dioxane | 49 |
| Octyltetraglycol | 46 |
| Iodide | 37 |
| Dithiothreitol | 34 |
| GOL | 34 |
| WO3 | 32 |
| aluminum fluoride | 32 |
| I42 | 31 |
| XENON | 25 |
| Methyltrioxitol | 24 |
| N-Cyclohexyltaurine | 20 |
| p-Mercuribenzoate | 20 |
| propylene glycol | 20 |
| MOPS | 19 |
| P6G | 19 |
| CPS | 17 |
| Lauryl maltoside | 16 |

Table S2: Summary of the IBIS predictions and CDD annotation validation for the 44 bound and unbound structures used as test set to compare with existing geometric approaches (metaPocket).

| PDB (of unbound structure) | Chain | Complex of the homolog | RMSD (Å) between complexed and unbound structures | Protein Description | Ligand Description (from the homolog) | Information of the inferred cluster containing the homolog | | | Total IBIS clusters | Valid clusters (only white clusters) | cd annoated biological clusters | no. of unique CD annots | Non-biological clusters (black) |
| --- | --- | --- | --- | --- | --- | --- | --- | --- | --- | --- | --- | --- | --- |
|  |  |  |  |  |  | **avg % identity of the cls** | **cdd annotation, if any** | **rank** |  |  |  |  |  |
| 1BBS | A | 1rne | 0.6 | Renin | NAG, C60 | 77 | catalytic motif | 1 | 11 | 9 | 4 | 3 | 2 |
| 1PSN | A | 1pso | 0.33 | Pepsin 3a | IVA, STA | 41 |  | 1 | 13 | 10 | 4 | 3 | 3 |
| 3LCK | A | 1qpe | 0.25 | Lck kinase | PP2, PTR | 46 | active site | 4 | 17 | 9 | 3 | 3 | 8 |
| 1CGE | A | 1hfc | 0.37 | Fibroblast collagenase | HAP | 61 | TIMP-binding surface | 1 | 7 | 3 | 3 | 2 | 4 |
| 1ESA | A | 1inc | 0.21 | Elastase | ICL | 59 | active site | 5 | 34 | 26 | 5 | 2 | 8 |
| 1HEL | A | 1hew | 0.21 | Acetylchitotriose | NAG | 84 | lysozyme catalytic cleft | 1 | 12 | 10 | 3 | 2 | 2 |
| 1HSI | A | 1ida | 1.41 | HIV 2 protease | QND, HPB, PY2, PPL | 51-56 | inhibitor binding site, catalytic motif | top 3 clustres | 8 | 6 | 3 | 2 | 2 |
| 1PDY | A | 1pdz | 0.54 | Enolase | PGA | 66 | substrate binding pocket | 1 (no ranking actually since all the clusters are singletons) | 4 | 1 | 1 | 2 | 3 |
| 1QIF | A | 1acj | 0.34 | Acetylcholinesterase | THA | 85 | catalytic triad | 2 | 32 | 17 | 5 | 2 | 15 |
| 2CBA | A | 2h4n | 0.33 | Carbonic anhydrase II | AZM | 92 | active site | 1 | 14 | 10 | 2 | 2 | 4 |
| 2CTB | A | 2ctc | 0.15 | Carboxypeptidase | LOF | 62 | active site | 1 | 8 | 2 | 2 | 2 | 6 |
| 2FBP | A | 1fbp | 0.89 | Phosphohydrolase | AMP, F6P | 94-96 | AMP binding site, active site | top2 | 6 | 5 | 2 | 2 | 1 |
| 2TGA | A | 1mtw | 0.31 | Trypsin | DX9 | 58 | substrate binding sites | 1 | 34 | 26 | 4 | 2 | 8 |
| 3P2P | A | 5p2p | 0.62 | Phosphilipase | DHG | 54 | catalytic network | 1 | 14 | 9 | 6 | 2 | 5 |
| 3PHV | A | 4phv | 1.28 | HIV 1 protease | VAC | 94 | inhibitor binding site | 1 | 9 | 7 | 3 | 2 | 2 |
| 3PTN | A | 3ptb | 0.26 | Beta trypsin | BEN | 59 | substrate binding sites | 1 | 34 | 26 | 4 | 2 | 8 |
| 4CA2 | A | 1okm | 0.34 | carbonic anhydrase II | SAB | 92 | active site | 1 | 14 | 10 | 2 | 2 | 4 |
| 5CPA | A | 7cpa | 2.17 | Carboxypeptidase | FVF | 62 | active site | 1 | 8 | 2 | 2 | 2 | 6 |
| 5DFR | A | 4dfr | 0.8 | Dihydrofolate reductase | MTX | 53 | folate binding site | 2 | 10 | 4 | 2 | 2 | 6 |
| 1HXF | H | 1dwd | 0.44 | Alpha thrombin + hirudin | MID | 55 | substrate binding sites | 1 | 38 | 28 | 5 | 2 | 10 |
| 1A6U | H |  |  |  |  | 61 | antigen binding site | 7 | 16 | 12 | 5 | 1 | 4 |
| 1GCG | A | 1gca | 0.32 | Galactose-binding protein | GAL | 95 | ligand binding site | 1 | 1 | 1 | 1 | 1 | 0 |
| 1IME | A | 1imb | 1.45 | Inositol monophosphatase | LIP | 87 | active site | singleton | 1 | 1 | 1 | 1 | 0 |
| 1NNA | A | 1ivd | 1 | Sialidase | FUC, ST1, NAG, MAN | 61 |  | top 2 clusters | 17 | 10 | 2 | 1 | 7 |
| 1STN | A | 1snc | 0.52 | Staphylococcal nuclease | PTP | 97 | Catalytic site | 1 (only one cluster) | 4 | 1 | 1 | 1 | 3 |
| 1YPI | A | 2ypi | 0.57 | Triose phosphate isomerase | PGA | 51 | substrate binding site | 1 | 13 | 4 | 1 | 1 | 9 |
| 1YPI | B |  |  |  |  | 51 | substrate binding site | 1 | 12 | 4 | 1 | 1 | 8 |
| 2SIL | A | 2sim | 0.25 | Sialidase (neuraminidase) | DAN | 100 |  | 1 (only one cluster) | 3 | 1 | 1 | 1 | 2 |
| 3TMS | A | 1bid | 0.24 | Thymidylate synthase | CBX, UMP | 71 | active site | 1 | 10 | 4 | 3 | 1 | 6 |
| 8RAT | A | 1rob | 0.28 | Ribonuclease A | C2P | 91 | catalytic site | 2 | 9 | 9 | 3 | 1 | 0 |
| 1AHC | A | 1mrg | 0.3 | Alpha momorcharin | AND | 47 |  | 2 | 14 | 7 | 0 | 0 | 7 |
| 1BRQ | A | 1rbp | 0.54 | Retinol binding protein | RTL | 96 |  | 1 | 3 | 1 | 0 | 0 | 2 |
| 1BYA | A | 1byb | 0.26 | Beta amylase | GLC | 62-77 |  | all four cls | 8 | 4 | 0 | 0 | 4 |
| 1DJB | A | 1blh | 0.23 | Methyl]phosphonate | FOS | 37 |  | 2 | 10 | 9 | 0 | 0 | 1 |
| 1IFB | A | 2ifb | 0.37 | Fatty acid binding protein | PLM | 57 |  | 2 | 4 | 4 | 0 | 0 | 0 |
| 1L3F | E | 2tmn | 0.62 | Thermolysin | PHO, NH2 | 96-100 |  | top 2clusters | 5 | 3 | 0 | 0 | 2 |
| 1NPC | A | 1hyt | 0.87 | Thermolysin | DMS, BZS | 71 |  | 1 | 5 | 3 | 0 | 0 | 2 |
| 1PHC | A | 1phd | 0.17 | Camphor 5-monoxygenase | HEM, PIM | 100 |  | top 2 (no ranking actually since all the clusters are singletons) | 7 | 2 | 0 | 0 | 5 |
| 1PTS | A | 1srf | 0.45 | Streptavidin | MTB | 84 |  | 1 | 8 | 3 | 0 | 0 | 5 |
| 1SWB | A | 1stp | 0.33 | Streptavidin | BTN | 88 |  | 1 | 8 | 3 | 0 | 0 | 5 |
| 1ULA | A | 1ulb | 0.61 | Purine nucleoside phosphorylase | GUN | 70-73 |  | top 2 (only two clusters in total) | 5 | 2 | 0 | 0 | 3 |
| 2CTV | A | 5cna | 0.44 | Concanavalin A | MMA | 97 |  | 2 | 8 | 3 | 0 | 0 | 5 |
| 2RTA | A | 1stp | 0.62 | Streptavidin | BTN | 86 |  | 1 | 8 | 3 | 0 | 0 | 5 |
| 8ADH | A | 1cdo | 1.17 | Alcohol dehydrogenase | NAD | 86 |  | 1 | 10 | 5 | 0 | 0 | 5 |

Table S3: Variety of ligands binding in the ATP binding pocket of tyrosine kinase homologs

| Ligand CID Name | PDB ID | # Inter. Res. | Interface Alignment |
| --- | --- | --- | --- |
| Imatinib | 1XBBA | 12 | ---L-S-----V-A--------------------MEMAE-GP--------------L-------- |
| STU | 1XBCA | 17 | ---LG------V-A-K-----------V------MEMA--GP-----------RN-L-SD----- |
| [C_20_H_23_N_5_O_2_S](javascript:SubmitMainForm('focus','168793');) | 3EMGA | 16 | ---L-S--F--V-A-K----E------V------MEMAE-G---------------L--D----- |
| [STU](javascript:SubmitMainForm('focus','168483');) | 1U59A | 15 | ---LG------V-A--------------------MEMA--GP-----------RN-L-SD----- |
| gamma-Imino-ATP | 2OZOA | 17 | ---L-CG----V-A-K------------------MEMAG-GP-----------RN-L--D----- |
| [thiophosphoric acid o-((adenosyl-phospho)phospho)](javascript:SubmitMainForm('focus','168504');) | 2G1TA | 16 | ---L-G---G-V-A-K------------------TEFM---N---------D-RN-L--D----- |
| thiophosphoric acid o-((adenosyl-phospho)phospho) | 2G1TD | 17 | ---L-G---G-V-A-K------------------TEFM---N---------D-RN-L--D----P |
| Imatinib | 2PL0A | 17 | -----------V-A-K----E-LM---V----I-T-YM----------IH------L-ADF---- |
| Bafetinib | 2E2BB | 26 | ---L----Y--V-AVK----E-VM-ILV----I-T-FM--G-----LFIHR------VADF---- |
| GIN | 2HZ0B | 20 | -------------A-K----E--M-ILV----I-TEFM---------FIH------LVADF---- |
| Imatinib | 1OPJB | 23 | ---L----Y--V-AVK----E-VM-I-V----I-T-FM--G------FIHR-------ADF---- |
| thiophosphoric acid o-((adenosyl-phospho)phospho) | 2G1TC | 15 | ---L-----G-V-A-K------------------TEFM---N---------D-RN-L--D----- |
| 9NH | 3B2WA | 19 | -----------V-A-K----E--M-LLV----I-TEYM--G---------------LIADF---- |
| Nilotinib | 3CS9A | 24 | ---L----Y--V-A-K---KE-VM-ILV----I-T-FM--G------F-H------LVADF---- |
| Imatinib | 2HYYD | 20 | ---L----Y--V-AVK----E--M-I-V----I-T-FM----------IH------L-ADF---- |
| Imatinib | 1OPJA | 20 | --L--------V-A-K----E-VM-I-V----I-T-FM---------FIH------L-ADF---- |
| Adenosine diphosphate | 2G2IA | 10 | ---GG------V-A--------------------TEFM---N--------------L-------- |
| thiophosphoric acid o-((adenosyl-phospho)phospho) | 2G1TB | 16 | ---L-G---G-V-A-K------------------TEFM---N---------D-RN-L--D----- |
| Nilotinib | 3CS9C | 21 | --L--------V-A-K---KE-VM-ILV----I-T-FM---------F-H-------VADF---- |
| Adenosine diphosphate | 2G2IB | 9 | -----------V-A--------------------TEFM--GN--------------L-------- |
| 7MP | 2HIWA | 23 | ---L----Y--V-AVK----E--M-ILV----I-TEFM--G--------H------LVADF---- |
| NSC735424 | 2FO0A | 19 | ---L----Y--V-AVK----E--M---V----I-TEFM--G---------------L-ADF---- |
| Imatinib | 2HYYC | 22 | ---L----Y--V-AVK----E-VM-I-V----I-T-FM--G-------IH------L-ADF---- |
| 4ST | 2HZ4B | 12 | -----------V-A-K------------------TEFM--G------------RN-L--D----- |
| Imatinib | 1IEPA | 20 | --L--------V-A-K----E-VM---V----I-T-FM--G-------IHR-----L-ADF---- |
| DB03878 | 1FPUB | 15 | -----------V-A-K----E--M---V----I-T-FM--G---------------L-ADF---- |
| 2,3-Diarylfuro[2,3-b]pyridine-4-amine, 8 | 2OF2A | 13 | ---L-------V-A--------------------TEYME-G------------AN-L--D----- |
| 7MP | 2HIWB | 22 | ---L----Y--V-A-K----E--M-ILV----I-TEFM--G--------H------LVADF---- |
| Nilotinib | 3CS9B | 24 | ---L----Y--V-AVK---KE-VM-ILV----I-T-FM--G--------H------LVADF---- |
| FMM | 3BBTB | 16 | -----------V-AIK-------M-----L--L-T-LM--G---------------L-TDF-L-- |
| FMM | 3BBTD | 14 | -----------V-A-K-------M---V----L-T--M--G---------------L-TDF-L-- |
| P3Y | 2QOHA | 17 | ---LG---Y--V-A-------------V------TEFMT-GN-----------R--L-AD----- |
| C_24_H_23_N_5_O | 2OF4A | 12 | ---LG------V-A---------------------EYM--G------------AN-L--D----- |
| DB03878 | 1FPUA | 17 | --L--------V-AVK----E--M---V----I-T-FM--G---------------L-ADF---- |
| NSC735424 | 1OPKA | 19 | ---L----Y--V-AVK----E--M---V----I-TEFM--G---------------L-ADF---- |
| PD 180970 | 2HZIA | 19 | ---L----Y--V-AVK-------M--------I-TEFMTYG---------------L-ADF---- |
| KIN | 2HZNA | 20 | -----------V-A-K----E--M-ILV------TEFM--------LF-H------LVADF---- |
| Dasatinib | 2GQGB | 19 | ---L-------V-AVK----E--M---V----I-TEFMTYG---------------L-AD----- |
| Nilotinib | 3CS9D | 19 | ---L----Y--V--------E-VM-IL-----I-T-FM--G------F-H------L-ADF---- |
| DIETHYLENE GLYCOL | 3BCEC | 7 | -----------------------M---V------TQ-M------------------L-T------ |
| Bafetinib | 2E2BA | 26 | ---L----Y--V-AVK----E-VM-ILV----I-T-FM--G-----LFIHR------VADF---- |
| Staurosporine | 1QPJA | 17 | ---LG------V-A-K----E-------------TEYM--GS-D---------AN-L--D----- |
| STU | 3D7TA | 15 | ---IG------V-A-K------------------TEYM--GS-----------R--L-SD----- |
| STU | 1BYGA | 16 | ---IGK-----V-A-------------V------TEYM--GS-----------RN-L-S------ |
| gamma-Imino-ATP | 2GS7A | 12 | ---LG-G----V-A-K------------------TQ-M---------------R--L--D----- |
| AEE788 | 2JIUA | 17 | ---L-------V-AIK----------------L-MQLMP-G--D-E----------L-TD----- |
